# *De novo* transcriptome assembly and analysis of gene expression in different tissues of moth bean (*Vigna aconitifolia*) (Jacq.) Marechal

**DOI:** 10.1186/s12870-022-03583-z

**Published:** 2022-04-15

**Authors:** Sandhya Suranjika, Seema Pradhan, Soumya Shree Nayak, Ajay Parida

**Affiliations:** 1 Institute of Life Sciences (ILS), An autonomous Institute under Department of Biotechnology Government of India, NALCO Square, Bhubaneswar, Odisha India; 2 Department of Biotechnology, Kalinga Institute of Industrial Technology (KIIT), KIIT Road, Patia, Bhubaneswar, Odisha India

**Keywords:** *Vigna aconitifolia*, Transcriptome assembly, Developmental stages, Differential expression

## Abstract

**Background:**

The underutilized species *Vigna aconitifolia* (Moth Bean) is an important legume crop cultivated in semi-arid conditions and is valued for its seeds for their high protein content. It is also a popular green manure cover crop that offers many agronomic benefits including nitrogen fixation and soil nutrients. Despite its economic potential, genomic resources for this crop are scarce and there is limited knowledge on the developmental process of this plant at a molecular level. In the present communication, we have studied the molecular mechanisms that regulate plant development in *V. aconitifolia*, with a special focus on flower and seed development. We believe that this study will greatly enrich the genomic resources for this plant in form of differentially expressed genes, transcription factors, and genic molecular markers.

**Results:**

We have performed the *de novo* transcriptome assembly using six types of tissues from various developmental stages of *Vigna aconitifolia* (var. RMO-435), namely, leaves, roots, flowers, pods, and seed tissue in the early and late stages of development, using the Illumina NextSeq platform. We assembled the transcriptome to get 150938 unigenes with an average length of 937.78 bp. About 79.9% of these unigenes were annotated in public databases and 12839 of those unigenes showed a significant match in the KEGG database. Most of the unigenes displayed significant differential expression in the late stages of seed development as compared with leaves. We annotated 74082 unigenes as transcription factors and identified 12096 simple sequence repeats (SSRs) in the genic regions of *V.aconitifolia*. Digital expression analysis revealed specific gene activities in different tissues which were validated using Real-time PCR analysis.

**Conclusions:**

The *Vigna aconitifolia* transcriptomic resources generated in this study provide foundational resources for gene discovery with respect to various developmental stages. This study provides the first comprehensive analysis revealing the genes involved in molecular as well as metabolic pathways that regulate seed development and may be responsible for the unique nutritive values of moth bean seeds. Hence, this study would serve as a foundation for characterization of candidate genes which would not only provide novel insights into understanding seed development but also provide resources for improved moth bean and related species genetic enhancement.

**Supplementary Information:**

The online version contains supplementary material available at 10.1186/s12870-022-03583-z.

## Introduction

There is a rising global demand for food legumes, which constitute a basic nutritional requirement [1]. Conventional food legumes are an integral feature of the crop ecosystem and although conventional pulse crops are grown on vast agricultural lands in both India and other parts of the world, food supply barely keeps up with demand. In addition, due to supply-demand imbalance, the conventional pulses are getting to be inaccessible for a sizable populace that can barely afford these crops due to their rising costs, thereby suffering acute malnutrition debilities [2]. This gap in supply-demand competition can be revoked by introducing, cultivating, and marketing some underutilized pulse crops that produce seeds with comparable dietary compositions to conventional ones [1, 3]. Contemplation towards such underutilized legumes is increasing for new alternate protein sources to meet all time increasing demand for vegetable proteins, particularly for the resource-poor families in challenging environments. Even though India is considered the center of diversity for Asiatic pulses, the knowledge about the genetic diversity and genomic divergence of some important species of local importance is largely limited.

Moth bean (*Vigna aconitifolia*) is an underutilized seed legume crop that has recently been identified as a potential supplement to the production of pulses for human consumption. This nutritious crop species is high in fiber and soluble proteins that contribute towards its health benefits. Moth bean has piqued the interest of researchers in developing countries to boost the genetic and genomic resources for this legume crop due to its high nutritional value and health benefits. It is classified as a plant in the Fabaceae family and is currently thought to be an ideal approach for cropping in arid to semi-arid areas of India [4]. India is a major producer of legumes, accounting for 29% of the global area and 19% of total production [5].

Moth bean is indigenous to India and Pakistan and is grown as a monoculture or as part of various cropping systems during the Kharif season due to its nitrogen fixation ability in conjunction with soil bacteria, early maturity, and relative drought tolerance [6], making it an ideal crop to grow in drought conditions due to its low input requirements [7]. It is a good source of nutrients, such as protein, starch, sugar, and minerals, with only a trace of anti-nutrients such as tannin, phytic acid, and lectins. It contains 22–24 % high-quality protein, as well as a high amount of essential amino acids, particularly lysine and leucine, and certain vitamins [8]. According to recent reports, there is a high concentration of secondary metabolites such as phenol and flavonoids. Catechin, which is abundant in moth bean, stimulates enzymes to remove free radicals and chelate metal ions. By inhibiting the gastric enzymes -amylase and -glucosidase, the seed extract has a promising anti-diabetic effect [3]. Moth bean seeds have medicinal properties as well and are used in diets to treat fevers. The roots are said to have narcotic properties. It also has trypsin inhibitors and antioxidant activity [9]. Trypsin inhibitors have a powerful anti-inflammatory effect and reduce the incidence of certain cancers [10].

Food and nutrition security is a critical issue not only in India but also in other countries around the world. The diet of the majority of India's population is deficient in some dietary essentials, resulting in a high prevalence of malnutrition. Pulses are relatively inexpensive sources of protein, making them valuable to people with lower incomes. The unique nutritional composition of moth bean will make it an excellent alternative for meeting the nutritional needs of the malnourished populations of a developing country. Underutilized food grains, such as moth bean, have a large potential for not only supporting commercially grown crops by reducing pressure on their availability, but they are also a cheap source of nutrients and can be raised at a low management cost [11].

As a result, moth bean is a low-cost, nutrient-dense species capable of mitigating the effects of drought stress and improving arid-region soil. To cover a wide range of transcripts in this study, we performed a comprehensive de novo transcriptome assembly of *Vigna aconitifolia* var RMO-435 based on the RNA Seq library. Tissue-specific transcript libraries were also studied to enable differential gene expression between tissues. This will assist in identifying the candidate genes involved in the growth, development, and metabolism of these legume crops.

## Materials and methods

### Plant Materials and growth conditions

Seeds of Moth bean (*V. aconitifolia*) var. RMO-435 was obtained from ICAR-CAZRI, Jodhpur, India and was grown under aseptic conditions in a mix of soil rite and vermicompost in a ratio of 3:1 in the greenhouse (16 hr./8 hr. light/dark; 65% RH) after being germinated on a moist filter paper for 24 hrs. Fresh leaf, root, and flower tissues were harvested from 30 days old plant and washed thoroughly with sterile water. The flowers were tagged on the day they opened completely. Young pods were collected at 5 DAA (days after anthesis) and seeds were collected at 10, 15, 20, 25 DAA intervals, frozen in liquid nitrogen, and stored at −80°C until use. The tissue samples for 10&15 DAA were pooled and labelled as early_seed and the tissue samples for 20&25 DAA were pooled and labelled as late_seed. Three biological replicates from each sample were used for sequencing.

### RNA extraction and sequencing

The method described by Shyamli et al., 2021 [12] was used to prepare libraries. Briefly, total RNA was extracted from 100mg of frozen plant tissues using Nucleospin Plant and Fungi RNA extraction kit (REF 740120.50, Macherey-Nagel, Düren, Germany) following the manufacturer’s protocol. The quantity and quality of the RNA were analyzed through Nanodrop 2000 Spectrophotometer (Thermo Scientific, Wilmington, DE, USA) and 1.2% agarose gel. 1μg of total RNA with RIN > 7 was used for library preparation. The libraries were prepared taking three biological replicates of each sample (Leaf, Root, Flower, Pods (5DAA)), early_seed (10 & 15DAA), late_seed (20&25 DAA)) using the TruSeq Stranded mRNA Library Prep kit (Illumina, San Diego, USA) following the manufacturer’s instructions. After quality assessment, eighteen libraries from three biological replicates representing six tissues were pooled and pair-end sequenced on the NextSeq550 (2x150) platform (Illumina, USA).

### Assembly normalization and quality assessment

The obtained reads were demultiplexed using the bcl2fastq software (Illumina, USA), and the raw reads of eighteen individual assemblies were filtered to remove low-quality reads and reads containing adapter sequences using the Trimmomatic- 0.39 software (ILLUMINACLIP: TruSeq3-PE130 2.fa:2:30:10 LEADING:5 TRAILING:5 SLIDINGWINDOW:5:10 MINLEN:50, [13] . After that, the high-quality reads were assembled using the Trinity pipeline (version 2.11) with the default parameters (http://trinityrnaseq.github.io/) [14]. The assembled contigs were merged with 98 % similarity using CD-HIT-EST (version 4.6.3) [15]. Transdecoder (https://github.com/TransDecoder/TransDecoder) was used to find the longest isoform for each gene (version 2.0.1) [16]. This considered as the final assembly and the assembled sequences are henceforth referred to as unigenes. The final assembled transcriptome's quality was determined by using the following parameters: (i) mapping back the clean reads onto the assembled transcriptome, and (ii) comparison with the Benchmarking Universal Single-Copy Orthologs (BUSCO) [17] database. In addition, indicators such as N50 and contig length distribution were used to assess assembly quality.

### Functional annotation

BLASTX (https://ftp.ncbi.nlm.nih.gov/blast/executables/blast+/2.9.0/) has been used to search a number of databases for putative functions for the assembled unigenes. All unigene sequences were aligned using BLASTx (*e* < 1e-5) to the following publicly available protein databases: UniProt Swiss-Prot (https://www.uniprot.org/), Pfam (http://pfam.xfam.org/), Eukaryotic Orthologous Groups of proteins (COG) (https://www.ncbi.nlm.nih.gov/research/cog-project/), and the Kyoto Encyclopedia of Genes and Genomes (KEGG) (https://www.genome.jp/kegg/kaas/). GO terms were assigned to the unigenes using an in-house pipeline.

### Differential gene expression analysis

The short reads from individual sample libraries (including replicates) were mapped onto the assembled transcriptome using Bowtie2 (http://bowtie-bio.sourceforge.net/bowtie2/index.shtml) [18] and abundance was calculated using RSEM (RNA-Seq by Expectation-Maximization-http://deweylab.github.io/RSEM/package) [19]. Differential gene expression studies was performed by using the statistical package Empirical Analysis of Digital Gene Expression (EdgeR) (http://biocon-ductor.org/packages/release/bioc/html/edgeR.html) with FDR correction ≤ 0.05, *P* value ≤ 0.001, fold change ≥ 2 [20]. EdgeR was also used to normalize the expected counts for relative expression and effective library size using the Trimmed Mean of M-values (TMM) normalization method. Differentially expressed genes (DEG) with threshold FDR ≤ 0.05 and log fold change (logFC) (≥ 2) were selected for further analysis.

### Identification of transcription factors

Plant TFDB (http://planttfdb.cbi.pku.edu.cn/download.php) was used to obtain peptide sequences for transcription factors from various plants. The NCBI BLASTX programme (ftp:/ftp.ncbi.nlm.nih.gov/blast /executable s/blast +/2.9.0/) was used to search *V. aconitifolia* unigenes against transcription factor sequences with an e-value cutoff of 10^-5^. MeV (v.4.8.1) software (https://sourceforge.net/projects/mev-tm4/) was used to generate all heat maps.

### SSR identification

The MIcroSAtellite (MISA) software (https://github.com/cfljam/SSRmarkerdesign/blob/master/misa.pl) was employed for identifying SSRs in *V. aconitifolia* unigenes using the following parameters in the misa.ini file: a minimum of 6 di-nucleotide repeats, 5 tri-nucleotide repeats, and 3 tetra, penta, and hexanucleotide repeats with a maximum interruption of 100 bases between two SSRs [21, 22].

### Quantitative real time-PCR

The relative expression of 15 genes was quantified using qRT-PCR according to the protocol described by Das et al., [23]. The FASTA sequences of the selected transcripts were retrieved and primers were designed using Primer3 online tool with the following criterion: amplicon size = 100–150 bp; primer length 18–23 bases; melting temperature of 57–63°C; and GC content of 40%–60%. For each RNA sample, 1 μg of total RNA was reverse-transcribed to synthesize cDNA using the first-strand cDNA synthesis kit (K1612, Thermo Scientific, MA, USA). The qRT-PCR was performed on QuantStudio-5 real-time PCR system (Thermo Fisher Scientific, USA) with SYBR green chemistry (Applied Biosystems, USA) in three technical and three biological replicates. *Actin* gene of *V.aconitifolia* was used as an endogenous control as we observed stable expression of this gene in all samples (Ct values were in the range of 19–21). Each reaction (5 μl SYBR Green, 1 μl template cDNA, 0.5 μl each of the primers (10μM), and 3 μl RNase-free water) was performed three times with the following program: 50°C (10 min), 95°C (3 min) followed by 40 cycles of 95°C (15 s), 58°C (1 min), and melt curve stage of 95°C (15 s), 60°C (1 min) and 95°C (15s). The relative expression was calculated using the comparative 2^_ΔΔCt^ method and taking leaf tissue as the control. The analysed data is presented graphically by taking the RQ values. The primer sequences for the unigenes are provided in Supplementary Table 1.

## Results

### Sequencing, de novo assembly, and assessment of assembled transcriptome

RNA-Seq libraries were constructed from six different tissues (leaves, root, flower, pod, early_seed, and late_seed) and sequenced on NextSeq 500/550 platform (Illumina) which produced about 520 million high-quality paired-end reads for three biological replicates for each tissue. The raw reads were filtered for quality and approximately 494 million clean reads (95%) were retained to generate the assembled transcriptome of *V. aconitifolia*. After removing redundancy and retaining the longest representative sequences, the final assembly consisted of 150938 unigenes with an N50 value of 1227 bp and with the largest unigene more than 16 Kb in length (Table 1). The majority of the unigenes were 500–1000 bp in length. The raw reads have been submitted to SRA database at NCBI bearing Accession number PRJNA788336.

**Table 1:** Assembly statistics of *V. aconitifolia* transcriptome

Attributes	Value
Total No of Unigenes	150938
N50 Value	1227 bp
N50 Index	35443
Total bases	141548001
Avg. size of unigene	937.78 bp
Length of Lagest unigene	16113 bp
Length of Smallest unigene	255 bp
GC%	46.23

We used three methods to assess the quality of transcriptome assembly of moth bean. First, all the clean sequence reads were mapped onto the assembled transcriptome of *V. aconitifolia* using Bowtie2. On an average, 92.33% of the reads could be mapped indicating a high rate of mapping onto the assembly. We then calculated and plotted the ExN50 values against the set of most highly expressed transcripts (Ex) [24]. This plot is a good indicator of whether the read-depth in a study is enough for a good quality assembly. A shift of the ExN50 peak towards >90% indicates good read-depth. In our study, we observed ExN50 peak at 97% which suggests that the reads used for the assembly are sufficient to generate full-length reconstructed transcripts (Supplementary Fig. 1B). Finally, BUSCO (Benchmarking Unique Single Copy Orthologs) was used to explore the completeness of transcriptome according to conserved ortholog content. The software was used to compare Moth bean transcripts with the database for Eukaryota (eukaryota_odb10) and Fabales (fabales_odb10). A BUSCO analysis was performed to evaluate the completeness of the *Vigna aconitifolia* transcriptome, recovering 240 of the 255 conserved eukaryotic genes (94.1%) and 4971 of 5366 conserved Fabales genes (92.6%) (Supplementary Fig. 1A).

### Functional annotation and classification

**Fig. 1:**
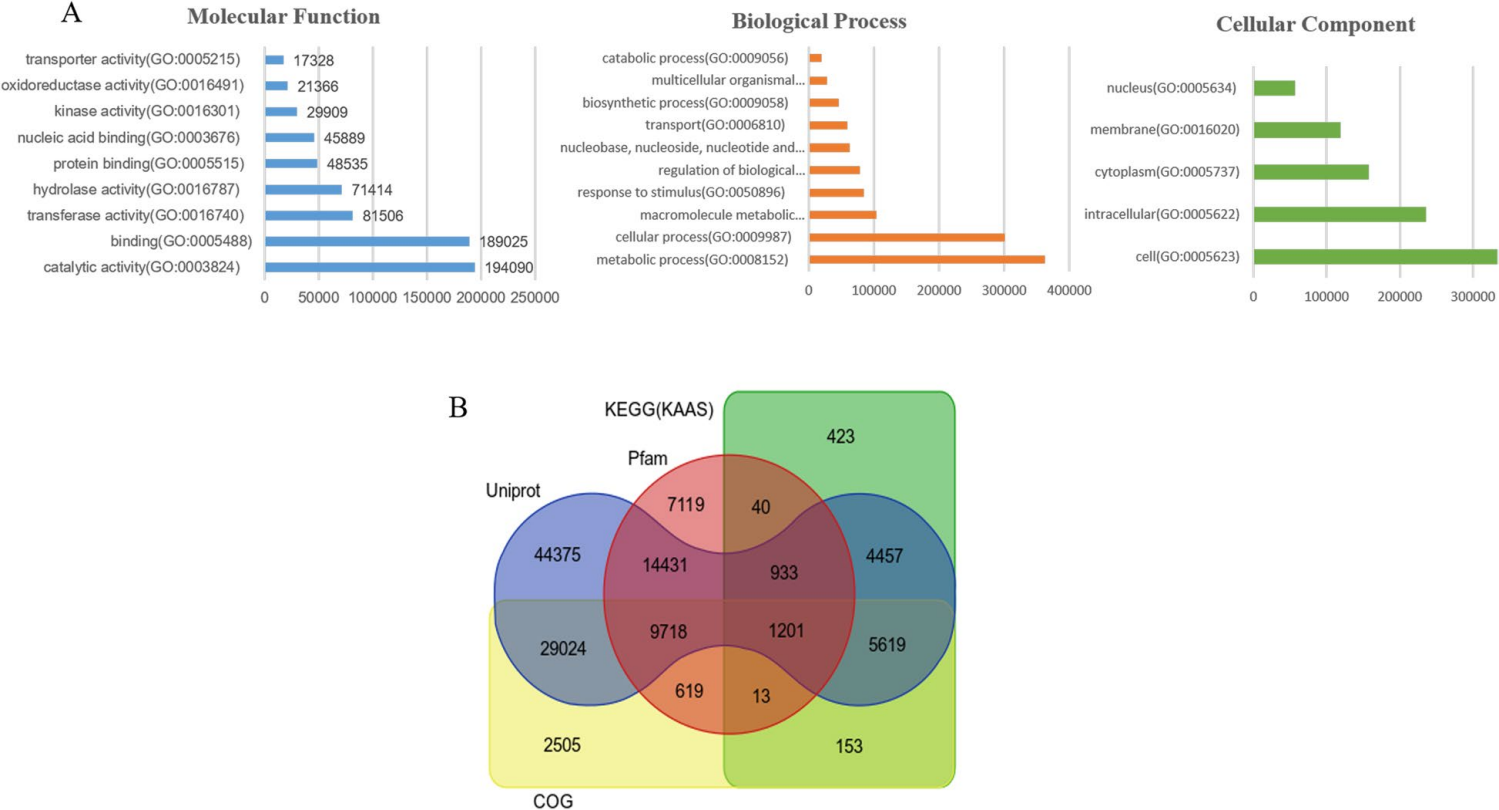
**A** Assignment of Gene ontology (GO) terms to predicted protein-coding genes of *V.aconitifolia*. **B** Venn diagram representing the annotation of protein-coding sequences of *V. aconitifolia* with various publicly available databases

The unigenes were annotated based on the UniProt, COG, KEGG, Pfam databases to decipher the general profile related to the biological functions represented in the transcriptome of *V. aconitifolia* (Fig. 1B)*.* In this study, 150938 unigenes were searched against the four databases and 120630 (79.9%) unigenes were annotated according to the databases. A total of 30308 unigenes did not significantly match the four public databases, which indicated that these unigenes might be novel transcribed sequences in *V. aconitifolia,* or else some unigenes were too short for statistically meaningful matches. The *V. aconitifolia* unigenes were assigned GO terms based on their annotation with the UniProt database and categorized into biological process, cellular components, and molecular function. Out of these, the majority were assigned to cellular components (332894; 36.17%), followed by biological processes (362369; 31.26%) and molecular functions (194090; 26.26%). Annotation based on GO terms (Gene Ontology) revealed that the majority of unigenes were categorized under “metabolic processes”, “cellular processes” and “macromolecule metabolic processes” under biological function (Fig. 1). In case of molecular function, most of the unigenes were found to have “catalytic” followed by “binding activity”.

### Differential gene expression

**Fig. 2:**
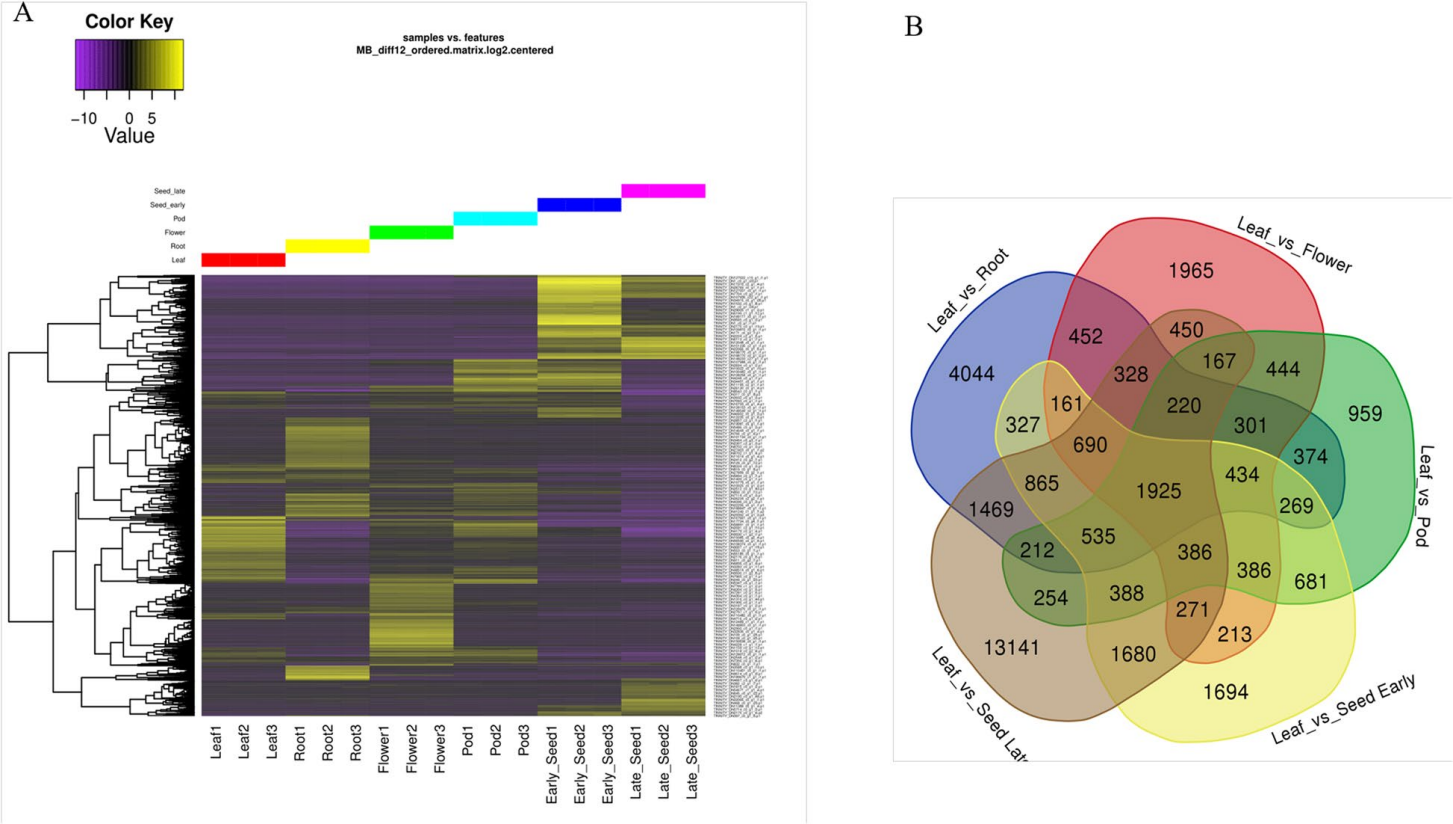
**A** Representative heat map of differentially expressed genes at various developmental stages. **B** Transcript abundance in different tissues as compared to leaf

We analysed the differential expression analysis of unigenes in various tissues of moth bean. *In silico* expression analysis revealed a total of 35,685 unigenes to be differentially expressed in different tissues as compared with leaf (*p*-value ≤ 0.001, FDR ≤ 0.05, fold change ≥2) (Fig. 2A). Of these, 1925 unigenes had differential expression in all tissues. A total of 4044, 1965, 959, 1694, and 13141 unigenes were differentially expressed in root, flower, pod, early seed stages, and late seed stages respectively, with leaf tissue as reference (Fig. 2B). We observed that a majority of the DEGs were up regulated in various tissues as compared to leaf (Table 2).

**Table 2:** Number of Unigenes differentially expressed in various tissues of moth bean with leaf tissue as reference

Tissue	Up-regulated	Down-regulated	Total
Root	3436	608	4044
Flower	1811	154	1965
Pod	744	215	959
Early Seed development	1113	581	1694
Late Seed development	8336	4805	13141

**Fig. 3:**
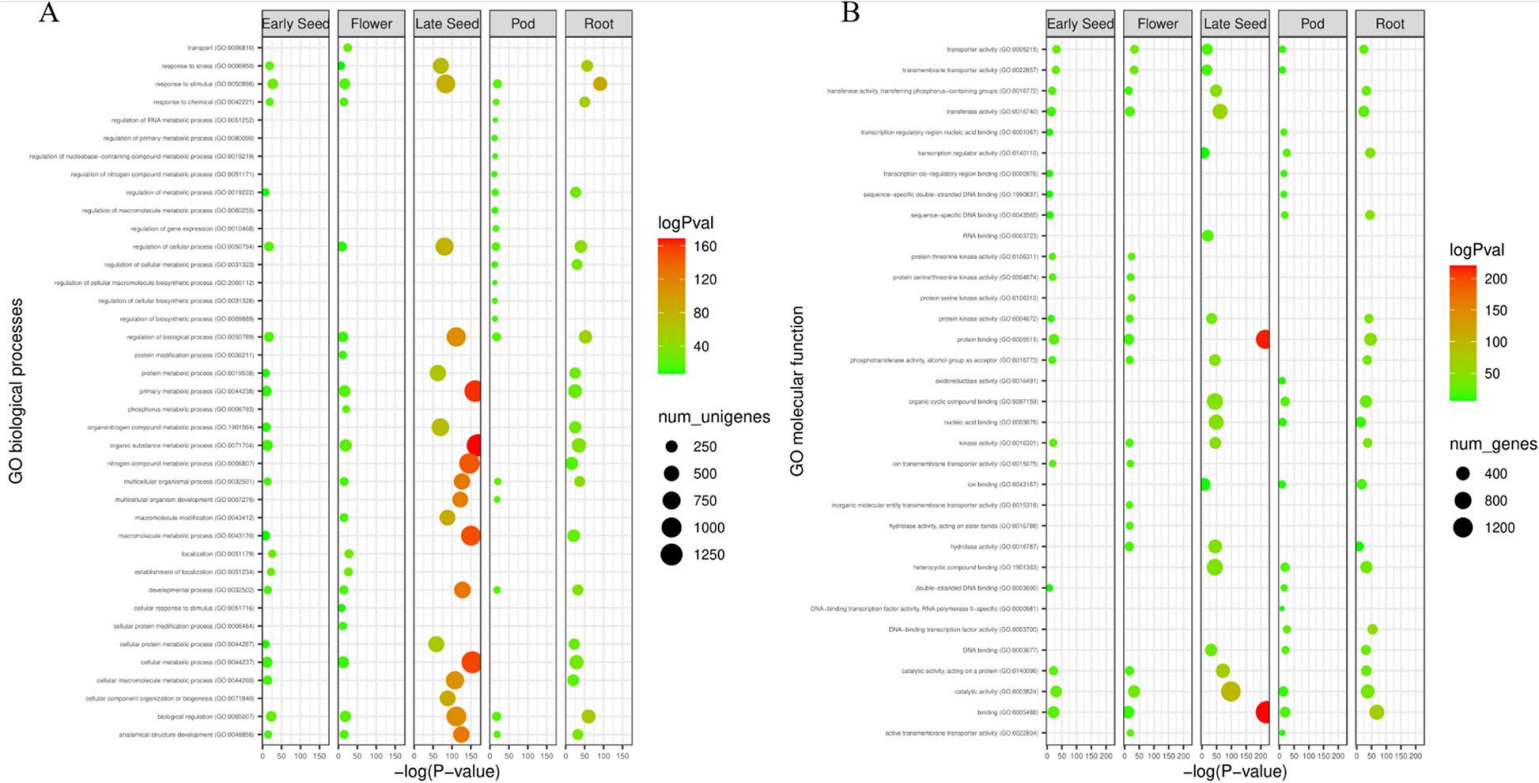
**A** Bubble plot of enriched GO terms associated with differentially expressed transcripts in different developmental stages for biological process. **B** Bubble plot of enriched GO terms associated with differentially expressed transcripts in different developmental stages for molecular functions

We analysed the GO term enrichment of the DEGs to get an idea of the biological processes and molecular functions that are overrepresented in the various tissues as compared to leaf tissue. Biological processes related to various metabolic processes such as “macromolecule metabolic process”, “nitrogen compound metabolic process”, “organic substance metabolic process” and “primary metabolic process” were enriched in late stages of seed development (Fig. 3A). We also observed a higher representation of GO terms for molecular functions such as “binding”, “catalytic activity” “RNA binding” and “transferase activity” in the late stage of seed development (Fig. 3B). We annotated the DEGs and found a number of genes to have high/low expression in various tissues of moth bean with respect to leaf tissue. There were a number of unigenes which showed significant differential expression in various tissues based on the parameters used for cutoff (FDR correction ≤ 0.05, *P* value ≤ 0.001, fold change ≥ 2) and consisted of TFs, receptors and transporters along with ribosomal proteins (Table 3).

**Table 3:** List of Differentially expressed unigenes with high/low expression in various tissues w.r.t leaf tissue

Tissue	Unigenes up-regulated with respect to leaf tissue	References	Unigenes down-regulated with respect to leaf tissue	References
Root	60S and 40S ribosomal proteins, WRKY transcription factors, MYB transcription factors, Putative disease resistance proteins	[25–27]	Pentatricopeptide repeat containing protein, Probable LRR receptor-like serine/threonine-protein kinase	[28]
Flower	Pentatricopeptide repeat containing protein, Subtilisin-like protease, WAT1-related protein, Peroxidases, Heavy metal-associated isoprenylated plant protein	[29]	ABC transporter	[30]
Pod (5DAA)	B3 domain-containing protein, ABC transporter family, Lipoxygenase, MYB, bHLH	[31–33]	ABC transporter, Zinc Finger protein, Phosphoglycerate kinase	[31, 34]
Early stages of seed development (10-15 DAA)	Beta-conglycinin, Vicilin-like proteins, bHLH, chaperone proteins Heat stress transcription factors, Receptor-like serine/threonine-protein kinase	[35, 36]	F-box proteins, WRKY Tfs, NAC Tfs, Pentatricopeptide repeat-containing protein, Protein NRT1/PTR FAMILY, CBL-interacting_serine/threonine-protein_kinase	[37, 38]
Late stages of seed development (20-25 DAA)	Pentatricopeptide repeat-containing protein, ABC transporters, Phospholipid-transporting_ATPase, Autophagy-related_protein, Zinc_finger domain containing_protein, F-box proteins, Beta conglycinin, chaperones, E3_ubiquitin-protein_ligase	[34, 39–42]	ABC transporters, AT-hook_motif_nuclear-localized_protein_1 bHLH Tfs, Zinc_finger_CCCH_domain-containing_protein, CBL-interacting_serine/threonine-protein_kinase, GATA Tfs, GDSL_esterase/lipase, Kinesin-like_protein, Nudix_hydrolase U-box_domain-containing_protein 60S and 40S ribosomal proteins.	[43]

### DEGs in *V. aconitifolia* flower and seed development

**Fig. 4:**
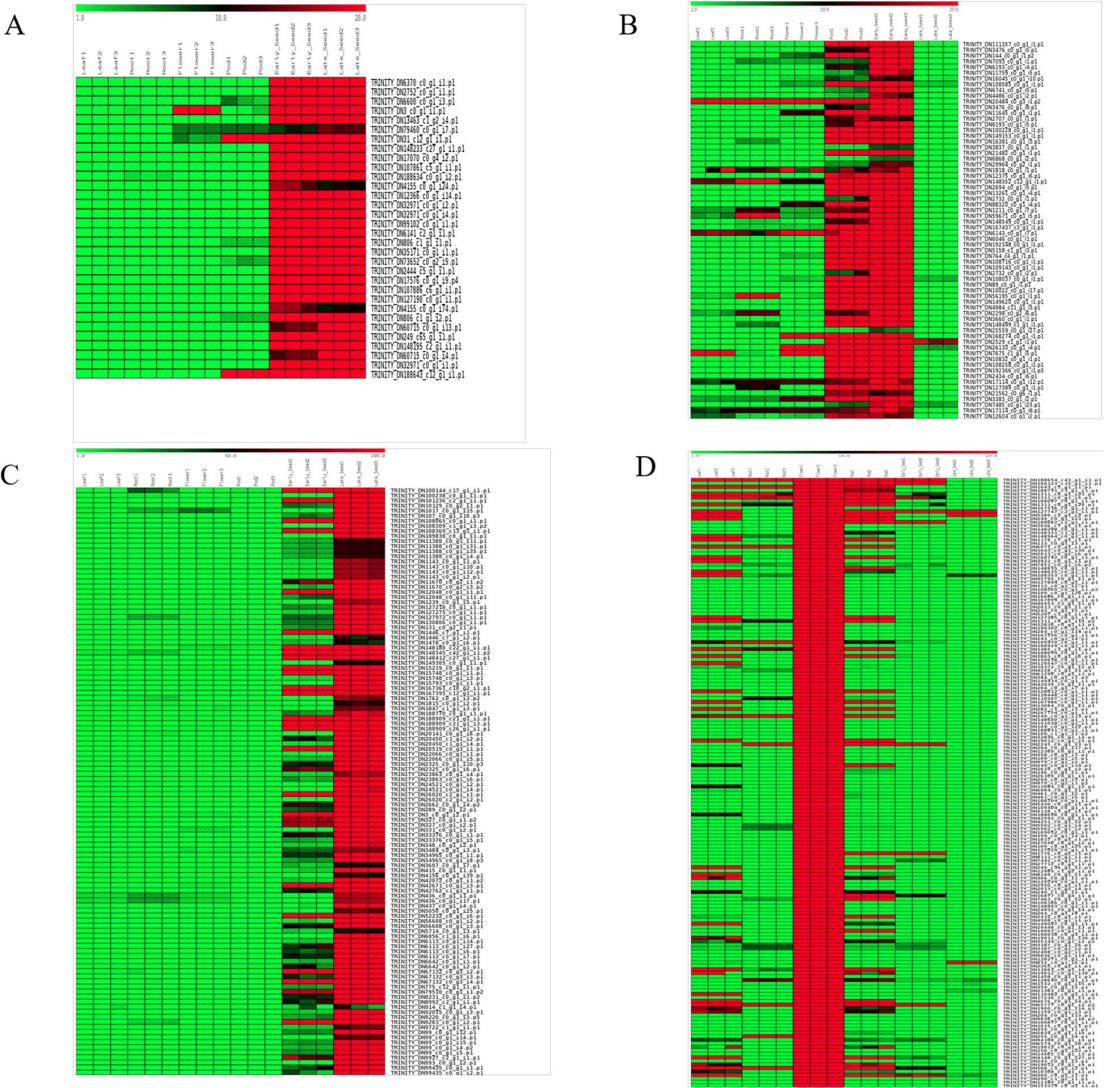
**A** Differential expression pattern of developing seed specific genes. **B** Differential expression pattern of young pod and early seed specific genes. **C** Differential expression pattern of late seed specific genes. **D** Differential expression pattern of flower specific genes

The young pods and seeds of moth bean used for human consumption are a good source of proteins, minerals, and also possess pharmacologically important qualities [3]. This implies that the genes regulating the process of seed development in moth bean are crucial to the quality and yield. Therefore, we analysed the unigenes that are differentially expressed in the reproductive stages of development in moth bean, namely, flower, young pods and developing seeds. We identified Agamous-like_MADS-box_protein and MYB like TFs to be up regulated in flower tissue in addition to genes encoding lipoxygenases, peroxidases and sugar transporters along with a number of pectinesterase/pectinesterase_inhibitors (Supplementary table 1, Fig. 4D).

We observed that genes encoding B3 domain containing transcription factor (ABI3), dehydration responsive proteins, lipoxygenases, sodium-hydrogen exchanger, abscisic acid responsive proteins (ABI5) were exclusively up-regulated in early and late stages of moth bean seed development (Fig. 4A, Supplementary table 1). A second set of unigenes had preferential expression in the young pods and early stage of seed development. It included genes encoding a bidirectional sugar transporter, a zinc finger CCCH domain containing protein, GDSL esterase/lipase, AP2/ERF TF among many others (Supplementary table 1, Fig. 4B). It was interesting to note that the expression of almost all of the unigenes in this cluster declined sharply in the late stages of seed development, suggesting that these genes are highly specific to very early stages of moth bean seed development. Similar analysis revealed an abundance of genes encoding late-embryogenesis abundant (LEA) proteins, heat stress transcription factors and seed maturation proteins in later stages of seed development in moth bean (Supplementary table 1, Fig. 4C). In addition to these, we also found genes for transporters, transmembrane channels and a few uncharacterised genes to have especially high expression in late stage of moth bean seed development.

### Identification of transcription factors

**Fig. 5:**
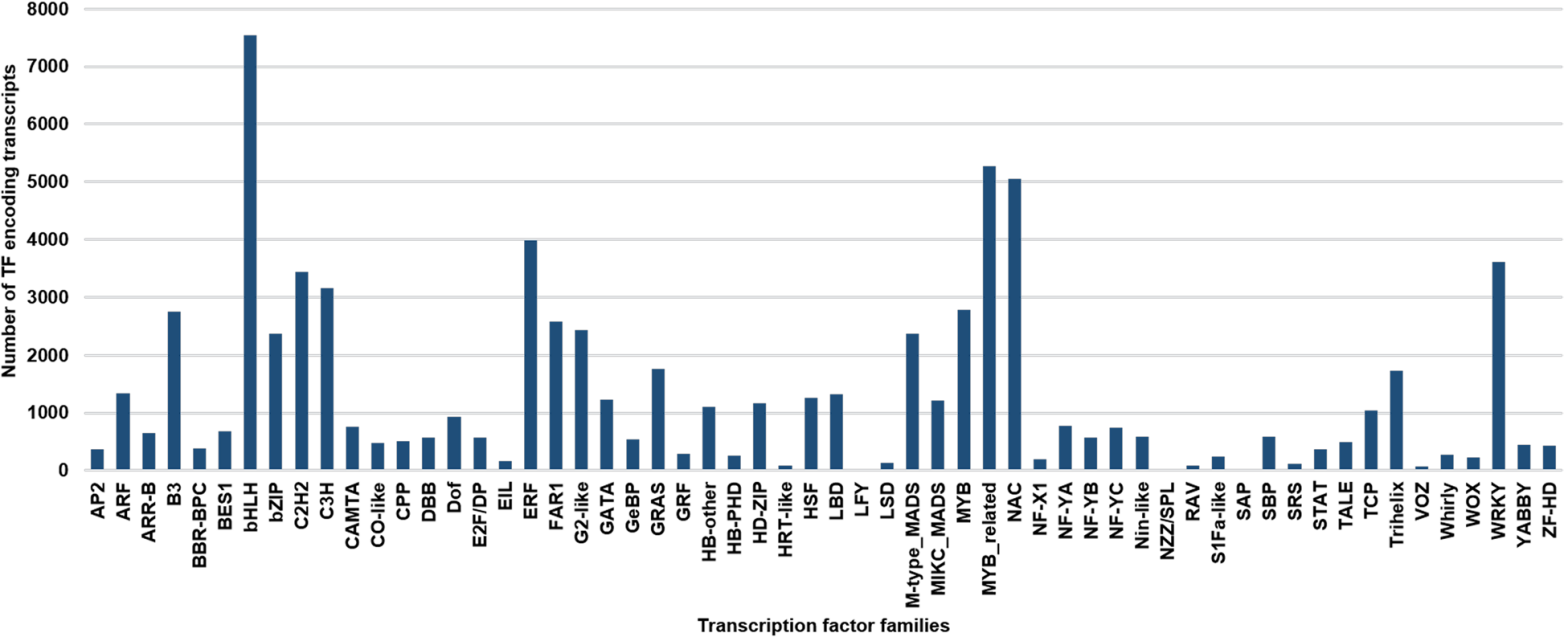
Genome-wide distribution of different transcription factor families in the Moth bean transcriptome. A bar graph representing Moth bean transcripts encoding transcription factors belonging to the most well represented families. Moth bean transcripts were subjected to BLASTx search against all the transcription factors in the PlnTFDB databases with an E-value cutoff of 1× 10^-5^

**Fig. 6:**
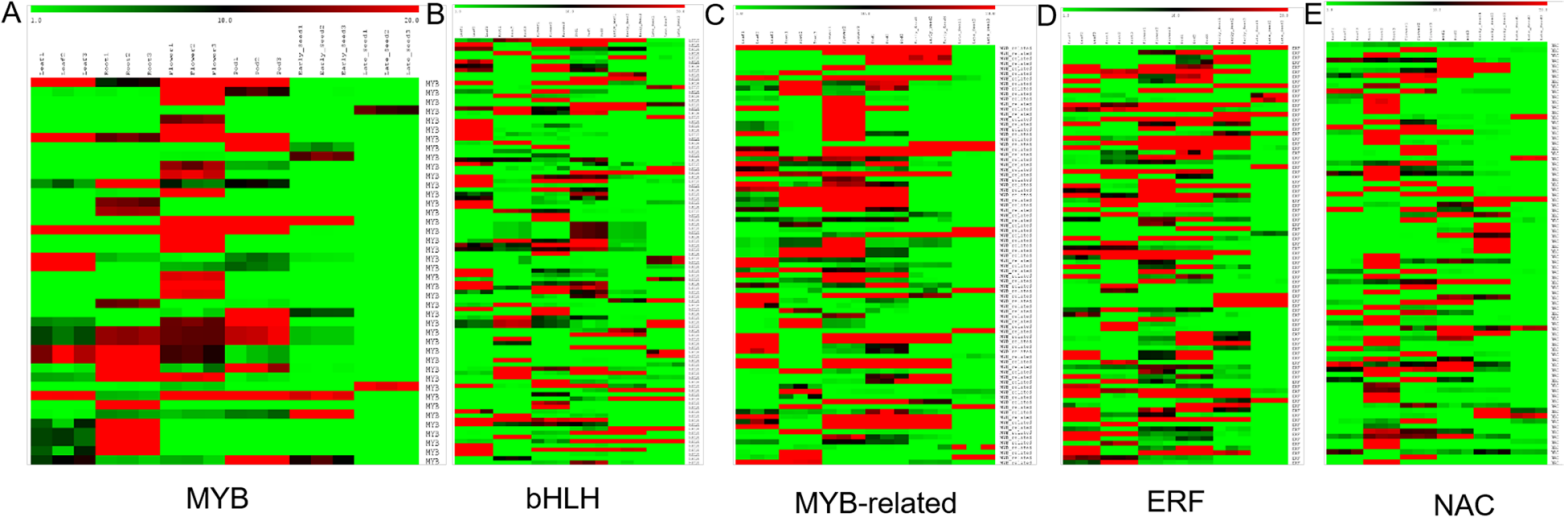
A heat map of differentially expressed Transcription factors in various tissues. **A** MYB, **B** bHLH, **C** MYB-related, **D** ERF, **E** NAC

Transcription factors are an important class of genes that regulate gene expression and control many aspects of plant growth and development [44, 45]. We conducted a homology based search of transcription factors in moth bean transcriptome and found a total of 74,082 unigenes to be annotated as various transcription factors. These were categorised into 58 families, of which, bHLH family of TFs was most abundant (7539), followed by MYB-related (5268), NAC (5055) and ERF (3933) and many others which are well represented in the transcriptome of moth bean (Fig. 5). *In silico* expression analysis of the TFs revealed that a number of them have significant differential expression (more than 6 fold) in various tissues of moth bean (Fig. 6, Supplementary Table 3). bHLH TFs were mostly upregulated in flower and young pods while NAC and ERF TFs showed higher expression in roots, flowers and young pods, early stage of seeds respectively (Fig. 6B, E, D). Apart from these TFs, other TFs like WRKY, MICK_MADS and Trihelix also showed basal level expression.

### SSRs identification

**Fig. 7:**
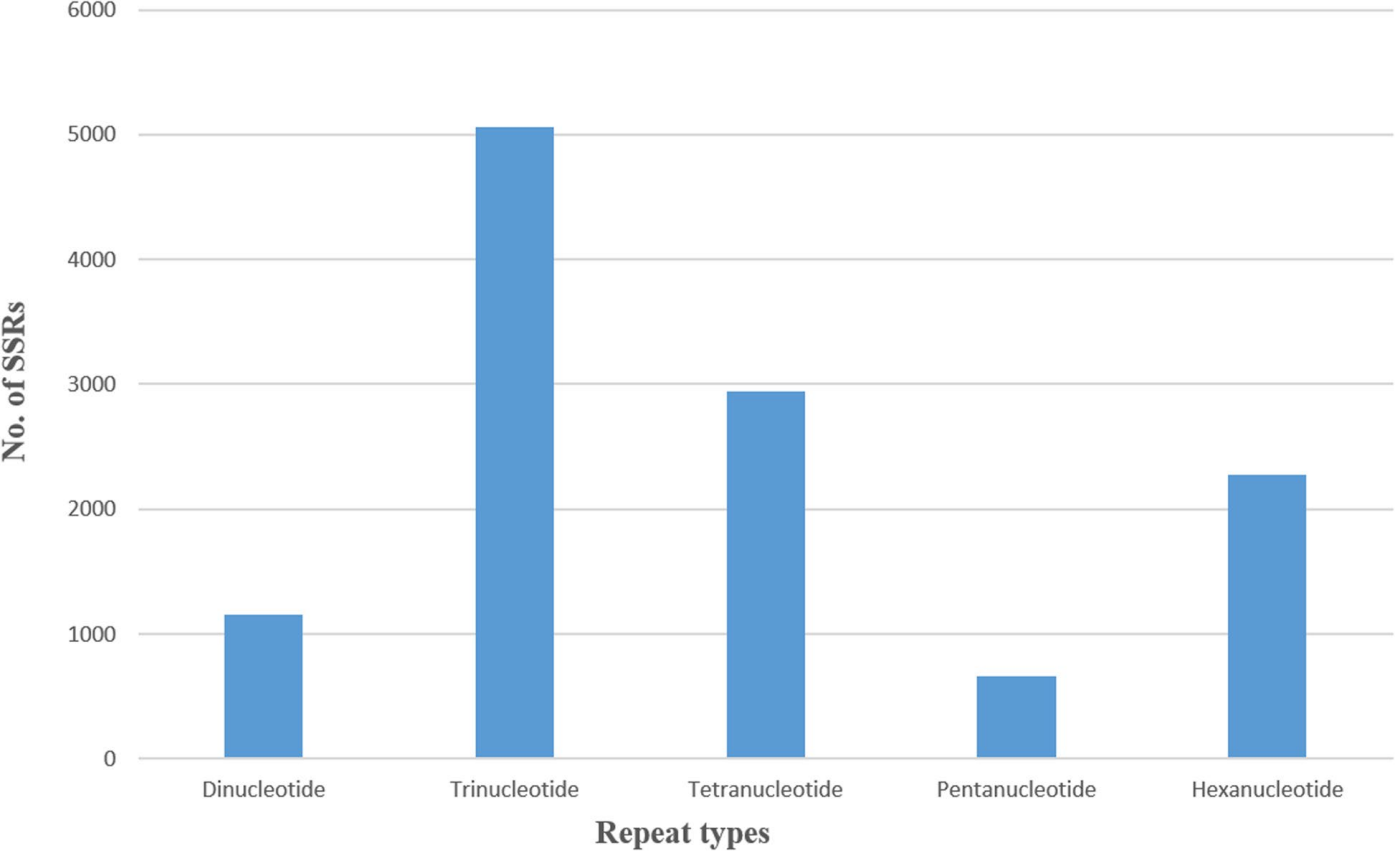
Distribution of SSR sequences in *V. aconitifolia* transcriptome

SSR (simple sequence repeats) markers are widely used because they are simple, rapid, low-cost, and repeatable. To overcome the problem of identification bias for non-gene regions, SSR markers based on target functional gene sequences or their upstream and downstream regions can be used to analyse diversity in molecular-assisted selection breeding [46]. SSRs are widely used in studies of genetic diversity and population structure of species [47, 48]. Out of the total 150938 contigs of the assembled transcriptome of *V. aconitifolia*, 12096 SSRs were identified, with 10597 containing SSR sequences (Table 4). Tri-, tetra-, and hexa-nucleotide repeats were the most common, followed by tetra- and hexa-nucleotide repeats (Fig. 7). Among the tri-nucleotide repeats, AAG/CTT repeats were found to be the most common SSRs. (See Table 4).

**Table 4:** Results of Microsatellite (SSRs) identifcation in *V. aconitifolia*

SSRs in *V. aconitifolia* transcriptome	
Total number of sequences examined:	150938
Total size of examined sequences (bp):	141548001
Total number of identified SSRs:	12096
Number of SSR containing sequences:	10597
Number of sequences containing more than 1 SSR:	1221
Number of SSRs present in compound formation:	728
Distribution to different repeat type classes	
Unit size	Number of SSRs
Dinucleotide repeats	1156
Trinucleotide repeats	5060
Tetranucleotide repeats	2942
Pentanucleotide repeats	663
Hexanucleotide repeats	2275

### Validation by qRT-PCR analysis

**Fig. 8.**
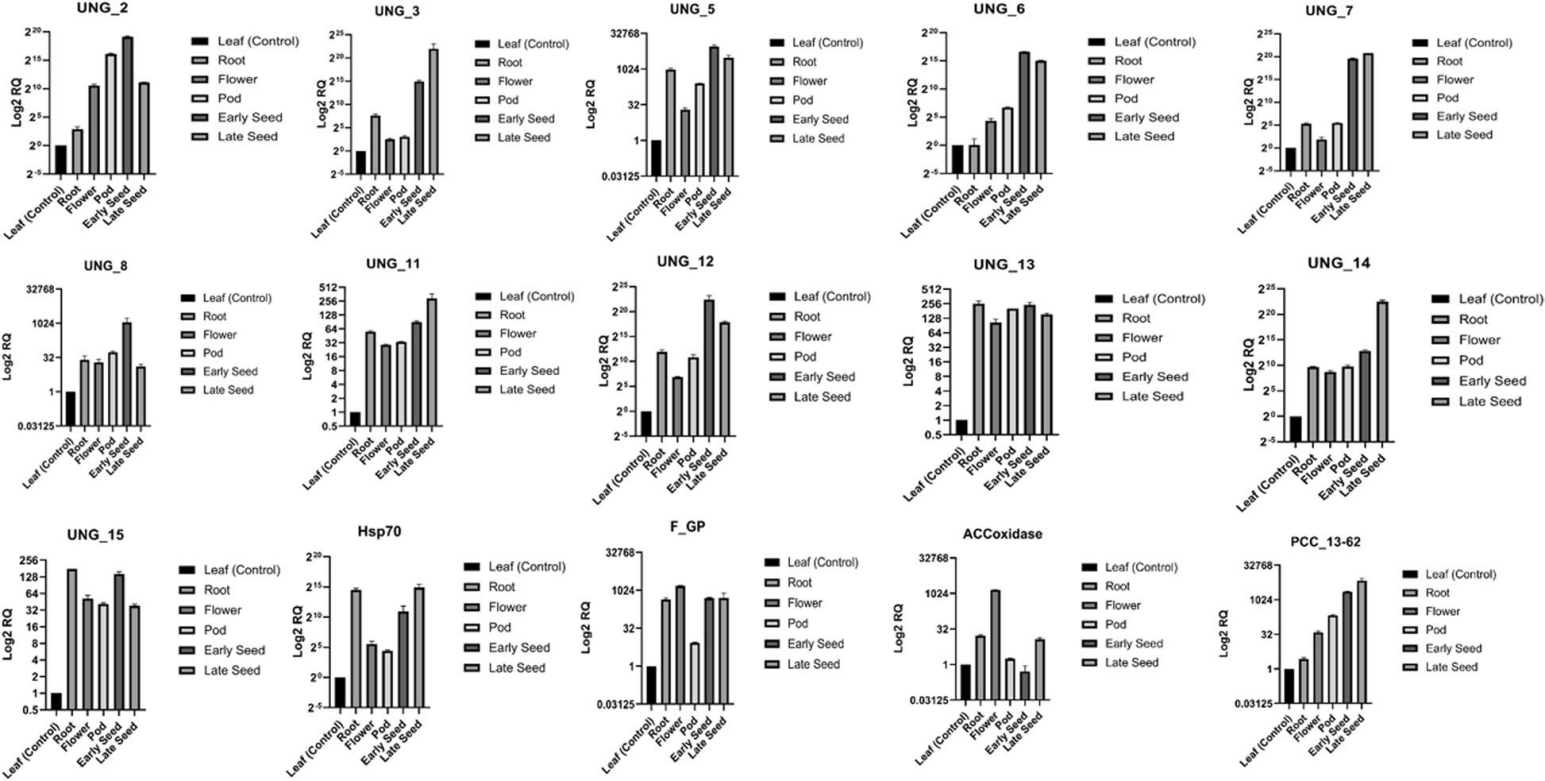
Validation of the expression patterns of 15 selected genes in 6 tissues by qRT-PCR

We carried out qRT PCR taking 15 candidate unigenes in different tissues. The selection of unigenes was done at random from *in silico* expression data. The qPCR analysis showed significant upregulation of Hsp70 (TRINITY_DN56042_c11_g1_i1.p1) in root and late seed tissues of *Vigna aconitifolia* (Fig. 8). UNG_2, 8 and 12 were upregulated in early seeds tissue and encode a bidirectional sugar transporter, a E3_ubiquitin-protein_ligase and a Late_embryogenesis_abundant_protein (LEA) respectively. UNG_3, 11, 14 and PCC 13-62 had higher expression in late seed tissue and coded for an embryonic protein, a Gibberellin-regulated_protein, Vignain and a Desiccation-related protein respectively. UNG_7, which encodes Phaseolin, was found to be up regulated in both early and late seed tissue with highest expression in late seed. Two of the unigenes, F-GP (encoding a gibberellin regulated protein, snakin2) and ACC (coding for aminocyclopropane-1-carboxylate oxidase) were found to have preferential expression in flower tissue while unigene UNG_15 (coding for an aquaporin) and a unigene coding for HSP70 had preferential expression in root and seed tissue. Unigenes UNG_5 (Beta-amyrin_28-monooxygenase), UNG_6 (Oleosin) and UNG_7 (Phaseolin) were up regulated in seed tissue of *V*. *aconitifolia*. Furthermore, the qPCR results revealed a strong correlation between the expression levels of the genes studied by qRT-PCR and their levels detected by RNA-seq (Supplementary Fig. 2, Supplementary data 1).

## Discussion

Underutilized legumes represent an untapped resource for meeting the dietary requirements of the resource-poor rural communities particularly, during drought and famine situations. The moth bean, a primitive crop of the genus *Vigna*, is valued for important agronomic traits like drought and heat resistance, which makes it an important legume that can grow in arid and semi-arid areas. The productivity of these crops has been low and there is a need for undertaking studies for genetic enhancement of these crops specifically for improving agronomic characteristics. Studying molecular mechanisms underlying plant development is an important step towards identifying important genes and pathways underlying complex biological processes. However, the generation and availability of genomic resources for moth bean lag significantly. Similar studies have been undertaken in model plants such as *Arabidopsis* [49] and *Medicago* [50], and few other species [51]. However, such studies in marginalised legumes, such as moth bean, are very limited. We have chosen to carry out the de novo transcriptome assembly of this nutritionally important but orphan crop, in order to augment the genomic resources and to facilitate whole-genome assembly through marker development.

Our analysis generated 150938 unigenes with an average length of 937.78 bp and N50 value of 1227bp. The quality assessment parameters indicated a good quality assembly which could be used for subsequent analysis. The assembly generated in this study displayed better attributes in terms of average length and N50 value when compared with other closely related legumes with published transcriptomes, including *Cicer arietinum* [52, 53], *Arachis hypogaea* [54], *Vigna radiata* [55] and *Cyamopsis tetragonoloba* [56]. Of the 150938 predicted unigenes, 120630 (79.9%) could be functionally annotated in the public databases, and *in silico* expression analysis revealed a total of 35,685 unigenes to be differentially expressed in different tissues as compared with leaf. Similar studies in another orphan crop Cluster bean (*Cyamopsis tetragonoloba* L. Taub), revealed 790 genes to be highly enriched in various tissues with as much as 50-fold higher FPKM value in one tissue compared to other. Majority of these (58.48%) were found to be specific to floral tissue followed by 22.15% and 19.37% in shoot and leaf [57]. Another transcriptome-based study in chickpea reported that majority of transcripts had preferential expression in root as compared with mature leaf, followed by flower bud [52].

GO term enrichment analysis was carried out for the DEGs and results showed that a number of biological processes like “metabolic processes”, “cellular processes” and “macromolecule metabolic processes” are over-represented in later stages of seed development. The enrichment of these GO terms re-iterates results obtained in similar studies where “metabolic processes and “catalytic activity” were over-represented [53]. However, these terms are broad and encompass a number of sub-categories, which become progressively specific to the gene activity. In order to get a more detailed insight into the gene activity, we analysed the DEGs in root, flower, pods, and seed tissues using leaf as control.

The analysis revealed that 60S and 40S ribosomal proteins are significantly up regulated in the root along with WRKY and MYB transcription factors. Similarly, Agamous-like_MADS-box_protein and MYB like TFs were upregulated in flower tissue in addition to genes encoding lipoxygenases, peroxidases, and sugar transporters along with a number of pectinesterase/pectinesterase inhibitors. However, differential gene expression analysis in our study showed that a number of kinases including LRR receptor like serine/threonine protein kinase, CBL receptor like serine/threonine protein kinase etc. were downregulated in various tissues of moth bean with respect to leaf tissue. Pentatripeptide repeat containing protein ABC transporters, TFs like MYB, bHLH, NAC etc constituted a significant proportion of the DEGs in all tissues indicating a diverse role of these genes in plant development [32, 33, 44]. Storage proteins were abundant in the stages of seed development along with a number of F-box proteins, E3 ubiquitin ligases, and chaperones, indicating a high rate of protein turnover in these stages.

TFs are known to play a role in abiotic stress and this has been well documented in legumes [58]. Previous reports suggest that TFs like MYB, WRKY, and bHLH families were mainly upregulated during abiotic stress and may be involved in the transcriptional regulation of flowering genes [59]. Many TFs have been found to play vital roles in plant growth and development, gene regulation, and function [44, 45]. In this study, the MYB family of transcription factors was the most abundant followed by bHLH, MYB-related, and NAC, ERF. In silico expression analysis of the TFs revealed that, a number of them have a significant differential expression (more than 6 fold) in various tissues of moth bean.

We carried out qRT PCR taking 15 candidate unigenes in different tissues. The qPCR analysis showed significant upregulation of Hsp70 (TRINITY_DN56042_c11_g1_i1.p1) in root and late seed tissues of *Vigna aconitifolia* (Fig. 8). UNG_2, 8 and 12 were upregulated in early seeds tissue and encode a bidirectional sugar transporter, a E3_ubiquitin-protein_ligase and a Late_embryogenesis_abundant_protein (LEA) respectively. UNG_3, 11, 14 and PCC 13-62 had higher expression in late seed tissue and coded for an embryonic protein, a Gibberellin-regulated_protein, Vignain and a Desiccation-related protein respectively. UNG_7, which encodes Phaseolin, was found to be up regulated in both early and late seed tissue with highest expression in late seed. Two of the unigenes, F-GP (encoding a gibberellin regulated protein, snakin2) and ACC (coding for aminocyclopropane-1-carboxylate oxidase) were found to have preferential expression in flower tissue while unigene UNG_15 (coding for an aquaporin) and a unigene coding for HSP70 had preferential expression in root and seed tissue. Unigenes like UNG_5 (Beta-amyrin_28-monooxygenase), UNG_6 (Oleosin) and UNG_7 (Phaseolin) were up regulated in seed tissue of *V*. *aconitifolia.* Moreover, the qPCR results showed that there was good correlation between the expression levels of the genes analyzed by qRT-PCR and their levels detected using RNA-seq (Supplementary Fig. 2).

Quantitative PCR revealed a number of unigenes with preferential expression in various tissues of moth bean plant. The up-regulation of genes for sugar transporters, E3 ubiquitin ligases and LEA proteins in early stages of seed development indicate biological activities like protein turnover are dominant at this stage. Similarly, the abundance of gene coding for storage proteins in the late stage of seed development in moth bean indicates that increase in seed size during the period of 20-25DAA. These observations confirm the expectations that early stages of seed development are marked by increased metabolic activity involving high protein turnover (synthesis and degradation), whereas the later stages were characterized by protein storage and nutrient reservoir activities. It was interesting to note that genes such as Hsp70, up regulated in root and early stages of seed development in moth bean. Hsp70 is known to have a role in abiotic stress tolerance and act as a chaperone to facilitate protein folding [60]. In addition, ACCoxidase and F-GP genes were showed to be upregulated in flower tissue. A similar trend has previously been reported in case of pineapple where ACCoxidase and F-GP have higher expression during flower induction [61]. Gibberellin and ethylene are phytohormones that are known to regulate both floral initiation and floral organ development. Previous research has identified and characterised those phytohormones in flower development. Furthermore, it was suggested that the ACCoxidase (*NsACO*) mRNAs accumulate rhythmically during flower development [62]. Another study found *CsACO2*, a member of the *ACO* multigene family, expressed in cucumber flowers [63]. Flowering is the primary developmental transition from the vegetative to the reproductive stage, and it necessitates genetic and epigenetic reprogramming to ensure seed production success [64]. This suggests that these complex networks of genes might play a role in the development of the moth bean flower. Further characterization and functional validation of these genes in moth bean (Var. RMO-435) will aid in unraveling their subcellular localization and cascade signaling pathways of this early-matured variety for the improvement of this nutritious plant.

Molecular markers identified through transcriptome-based studies are known to be genic in nature and thus are expected to be exceptional choice in molecular breeding applications. Because of their high amplification rates and high cross-species transferability, transcriptome-based markers are more useful than non-transcribed region markers [65]**.** Though SNPs are the markers of choice for understanding the trait architecture [66], for breeding application, SSRs and other gene/length polymorphism based markers are preferred. A total of 12096 SSRs were identified of which 10597 contained SSR sequences out of the total 150938 contigs of the assembled transcriptome of *V. aconitifolia*. The largest fraction of SSRs identified were trinucleotides (41.8%) followed by tetra nucleotides (24.3%), as also reported by several studies in other plants [67, 68].

## Conclusion

In this study, we have produced the first large-scale transcriptome sequence of various developmental tissues of one of the underutilized legume species lacking genomic resources. Functional annotation of the transcriptome provides a general perception of the gene content, biological processes, and pathways that operate in moth bean to provide the plant with its specialized functions. The identification of tissue-specific transcripts lays the foundation for accelerating the functional analysis of genes of interest in moth bean. The data and inferences generated in this study provide essential information for future genetic studies in moth bean. The resources generated here will prove to be essential to pave the way for functional and comparative genomic studies of this promising nutritional and nutraceutical plant in the future.

## Supplementary Information

**Additional file 1:** Supplementary data.

**Additional file 2:** Supplementary Fig 1a.

**Additional file 3:** Supplementary Fig 1b.

**Additional file 4:** Supplementary Fig 2.

**Additional file 5:** Supplementary Table 1.

**Additional file 6:** Supplementary Table 2.

**Additional file 7:** Supplementary Table 3.

## Data Availability

The raw data presented in the study is available in the NCBI SRA database under Bio Project PRJNA788336, Accessions SAMN23965025-SAMN23965042.
