# Supplementary material for: De novo transcriptome assembly and analysis of gene expression in different tissues of moth bean (Vigna aconitifolia) (Jacq.) Marechal
Source: BMC Plant Biol. 2022 Apr 15;22:198. doi: 10.1186/s12870-022-03583-z (PMC9013028; doi:10.1186/s12870-022-03583-z)
Supplement: Supplementary file 1 — Additional file 1: Supplementary data. [file 12870_2022_3583_MOESM1_ESM.pdf]

Supplementary data 1: Fasta Formatted nucleotide sequences of unigenes selected for qRT PCR

>TRINITY_DN100228_c0_g1_i1.p1 ((UNG_2)

CATTACGCACTATTACCAAGGCAAAATGGTTTTTCTTTTTTTTTTTTTCACTTGTGTATA
AATAACTGGAGTTTCAACAACACATCCTCATTCCCAAACCTGCACCAGATTGAGAGATTG
AGAGGGTTGCTTTGGGATAAACCCTCACAACACCTCCAATCATATCATATCATGGCCATC
TTGGGTAGTCATAATCCTCTCGCAGCCACTTTTGGCATATTTGGAAACATCATTTCCGTT
ATGGTATACCTGGCTCCAGCGCGAACGTTTCGGAAAATCTACAAAAAGAAATCCACCCAA
AGTTTCCAATGTCTACCTTACTTGGTGGCATTATTCAGCTCTTCACTTTGGTTGTACTAT
GCATCATTCAACATCAAACATGCCATCTTGCTTGTCTCGATAAACTCCTTTGGATGTGTG
ATAGAGATCATCTACATTGTCATCTTCATAAAGTATGCGGATAAGGATGCCAAGAGATTA
ACCATTAAGCTACTTGCTGCAATGAACTTTGGGTCCTTGGCCTTGATCGTTTTGGTCACG
CGTTTCGCCATCGATGAGTCCTATCAAGTGAAAGTCCTCGGATGGGTTTGTGACGTGGTT
TCAGTGATTGTATTTGCAGCACCTCTTAGCGTAATGTTACAAGTTATAAGAACGAAGAGT
GTACAATTTATGCCCTTCTGTTTGTCATTTTCCCTCTTACTGAATGCCATAATGTGGCTT
GCTTATGGTTTCTTCAACAAGGACATGTGCGTTGCCCTGCCAAATGTGGGGGGTCTAGCA
CTGGGACTGCTTCAGATGTTGCTACATGCCATTTACAGGAATCGTGGTGCGAAGGAGAAC
GTAACGACAGACGGTGCAGTGACAACCTTTGTGGTGGTCGTGAATCCATCGGGGCCTCCA
GAAGTGTTCTCAACAGCAGTGAAAGATGAACTATCACTGGAAAATAATAAAGGTGGAGAA
GACGATGCACAAGGGAAAACCGTGGAAGCCAACGATTGCCCTCTTTGA

>TRINITY_DN108369_c13_g1_i1.p1 (UNG_3)

ATGGCGTCGGGACAGCAATTCAAGGGAGACAGAGCGGAGGCAGCGGCCAAACTTGCAGCC
AAAGACATCGGCGATATCAACAGAGCGAACGAGCGCAACGACAACTTCGACCTTCATTCC
GAGGCCAACTTTAAGCAAGCACGAGCAGAAGCAGCTGCGAAACTTGCCGCAAAGGATCTG
GAGGATGTTAACAGAATGAGGGACAACAATGCCACCACATTCAAAGAGCAGCAGCACTAC
GGCGAGAACAGACCCGGTGTGATAGGGTCCATGTTCAGAGCCGCTAAGGAAGCGGTGGTT
GGGAAGCCTCGCGATACGGTTGAGACCTGTCCAGGGGTTTACGATTCCAGCAGAGAGAGG
ACAACGAAGGAGCAAGGTGGGTCGAAGATGGGGGAATATGCAGATTATGCGACCCAGAAG
GCGAAGGAAACGAAGGATGCAACGGTGCAGAAGGCTGGGGAGTACACAGACTTTGCGACT
CAGAAAGCAAAAGAAAGCAAGGACTATGCGGCAGATAAGGCAAAGGAAGCTAAAGATGCT
ACAGTGCAGAAAGCTTCAGAGTACAAGGATTATACTGCTGAGAAGGCTAGGGAAGGGAAG
GACAGTGCAACGGGAAAGCTTGGGGAGTTGAAGGACTCTGCTGCTGATGCTGCTAAAAGA
GCTATGGGCTACTTCACAGGCAACAGAGACGAAACAAGGGTGGGTGGAACGGCGGAGGAT
ACCAGGCGAAGAACCCAAGAGGTGAGGGTGCAGGATAAGGACTATGGAACGGGGTATGGA
GGAGAGAAGGTGGTGATAAAAATGGAAGAGTCTCGGCCGGGAGCGGTGGCGGATGCGATG

AAAGCGGCGGAGAGGGCCTTGGGCGGCGAGATGGAGGAAGAAGGTATCCTTCGTGTGGAG
CGTCGTAGGGAGAAAATGTGA

>TRINITY_DN148549_c0_g1_i1.p1 (UK_5)

ATGGAGCATTTCTATCTTTCCCTCCTTCTCCTCTTTGTTTCTCTCGTCACCTTCTTCTTC
TTCCTCCTTTTCCACAAGCACCGCTCTCCCTTCTCCGCCCCCAACCTCCCACCGGGAGCC
ACCGGTTTCCCGCTCATCGGGGAGAGTCTCCAGTTTCTCTCCACCGGATGGAAAGGTCAT
CCGGAGAAGTTCATTTTCGACCGCATGATCAAGTACTCCTCCAACCTCTTCAAAACCTCC
ATCCTAGGGGAACCCGCCGTGGTGTTCTGCGGCGCCGCCTCCAACAAGTTTTTGTTCTCA
AACGAGAACAAGCTGGTGGCGGCGTGGTGGCCGGAGAGCGTGAACAAGGTGTTCCCCACG
ACGGTGCAGACGAACTCGAAGGAAGAGTCGAAGAAGATGAGAAAGTTGCTCCCACAGTTC
CTAAAACCCGAGGCTCTCCAACGCTACGTTCCCATCATGGACAACATTGCACAGACCCAC
TTTTCTTCCCTTTGGGACAACCACACCCATCTCACCGTCTATCCCTTGGCCAAAAGGTAC
ACATTTATGTTGGCGTGTCGTTTGTTTATGAGCGTTGAGGATGTGAATCACGTAGCGAAA
TTTGAGAACCCTTTTCATCTTTTGGCGGCCGGAATCATATCAGTGCCCATCGATCTTCCC
GGAACGCCGTTCTACAAAGCTATCAGAGCAGCAAAAAGCATCAGAAAGGAGCTGTTGAAG
ATCATCAGAGAGAGGAAAGATGCTTTGGGCCAAGGAAATGCTTCCCCAACACAAGACATT
TTGTCTCACATGCTGCTCACGTGCGATGAGAACGGACAGTTCATGACTGAATTGGACATT
GCAGACAAGATTCTCGGACTTTTGATCGGTGGTCATGACACTGCAAGTGCTGCATGCACT
TTCGTCGTCAAATACCTCGCTGAACTCCCTCACATTTATGATGCAGTCTATCGAGAGCAA
ATGGAAATTGCAAAGTCGAAATTGCCGGGAGAGTTATTGAATTGGGATGATATAAACAGG
ATGAAATATTCTTGGAATGTAGCTTGTGAAGTGATGAGAATTGCTCCTCCACTTCAAGGA
GGTTTTAGAGAAGCTATCAATGACTTTACTTTCAATGGCTTCTCAATTCCAAAGGGATGG
AAGTTGTACTGGAGTGCAAATTCAACACATAAAAGTCCAGAATACTTTCGAGAACCAGAG
AAATTTGATCCAAGCAGATTCGAAGGAGAAGGACCAGCTCCTTACACTTTTGTGCCATTT
GGTGGAGGACCAAGAATGTGCCCTGGAAAAGAGTATGCAAGGTTGGAAATCTTGGTTTTC
ATGCACAACTTAGTGAAGAGGTTTAAGTGGGAAAAAGTTATTCCAGATGAAAACATCATC
GTTGATCCCTTACCCATTCCTGCAAAAAACCTCCCAATTCGTCTTTATCCTCACAAACCC
TAA

>TRINITY_DN189069_c6_g1_i1.p1 (UNG_6)

ATGAGTGATCGCACACAAGCACCCCACCATGTCCAGGTCCACACCACAACCCAACACTAC
GAAGCTGGTGTCGTTCCTCCAACTCGATATGAGCGTGCTGTTTTTCCCCCACAGTTTTAT
GAATCTGGAGGTAAGACTAGATCTTACCCTGCCGAGAGAGTTCCCTCCGCCACCCAGATC
CTCTCTGTTGTCGTCGGCCTTCCGGTCGGCGGCCTCCTCCTTCTTCTGGCCGGACTTACT
CTGGCAGGAACTCTAACTGGGCTGGCGGTGGCCATCCCGCTCTTCATTCTGTTCAGCCCA

GTTCTGGTTCCAGCTACCGTGGTAATCGGGCTGGCAGTGGCGGGGTTTTTGACGGCCGGA
GCTTTCGAGCTGACGGCTGTGTCGTCGTTCTCGTGGATCTTGAATTACATCCGGGAGAGC
CAGGGGCCAGTGCCGGTGAACCTGGCGGTAAAGGCGAAGCATCAGCTGGCGGATGCTGCA
GAATACGTGGGGCAGAAGACAAAGGAAATGGGACAGAAGACAAAGGAAGTTGGGCAGGAT
ATACAGAACAAGGCTCAGGATGCAAAGGAAAAGAGTCTGAAGGAAGCCAAAGATGCAAAG
GAGATGAAGAGAACAACAGTAACAGCAACAGCCGCAACAGACTGA

>TRINITY_DN28769_c0_g1_i1.p1 (UNG_7)

AGAGCAAGGATTCCACTGCTGCAGTTGCTGGGAATTCTTTTACTGGCATCACTTTCTGTC
TCTATCGGCATTGTCCACCGGGAGCACCTAGAGAGCCAAGAAGAGTCTGATTCAAGAGGA
CAAAATAACCCCTTCTACTTCAGCTCCGACAGGAGATTCCACACTCTATTCAAAAACCAA
TATGGTCATCTTCGGGTCCAACACAGGTTCGATAAACGCTCCAAACAAATTCAGAATCTT
GAAAACTACCGTGTCATAGAGTTAAAGTCCAAACCCAACACCCTCCTTCTTCCTCACCAT
GCTGATGCCGATTTC

>TRINITY_DN3383_c0_g1_i2.p1 (UNG_8)

ATGGCGCAGCTTCCCTGTGACCCAGAAGGCGTTTGCATGCGATGTAAGGAGAAACCCCCT
TCTTCGCAAACCCTCACCTGCCGGACCTGCGCCACTCCATGGCACCTTTCTTGCCTCACT
GAGCCTCCGGTCTCCGATTCCGACACCCACTGGGAGTGTCCCGACTGCTCTGACCTCTCC
AATCCTGTTCCCGTCGCTCCCTCCGACGGCATTCTCCTCGCCGTCCGCGATATTCAGTCA
GACACGTCACTCTCCGAAAAACAAAAGGCAAAAAAATGCCAAGAAGTGCTCGCCGGTTCC
TCCGATCTGTCCAAGGACAAAAATGCTCCCGATATATTCGATGGGAGTTTCAACTGCTCT
ATCTGCATGCAACTGCCTGATAGGCCTGTCACTACACCTTGTGGGCATAATTTCTGCCTG
AGGTGCTTTGAGAAATGCATCAATCAGGGGAGGCATAATTGTGCGAATTGTCGGACTCCA
ATCCCTTCAAAGATGTATAGTCAGCCGAGAATTAACTCTCAGTTGGCTATGGCTATTCGA
TTGGCTAAGGCAGCGAGGTCTGGGCAGTCTTCTGGGCCACCTAAAGTGCATCACTTTGTG
CGCAATCAGGATCGCCCAGATACTGCCTTTACCACTGATCGGGCGAAGAAAACTGGAAAA
GCTAATGCTTGTTCTGGTAAGATTTTTGTTACGGTTCCACCCGACCATTTTGGGCCAATT
CCAGCTGAAAACGATCCCAAGAGGAATCGCGGGGTTTTGGTTGGTGACACGTGGGAGGAC
AGGATGGAATGTCGGCAATGGGGCGCTCATTTACCTCATGTAGCTGGTATCGCTGGACAG
AGTAGTTATGGTGCCCAATCTGTTGCTCTCTCGGGTGGTTACTTGGATGATGAGGATCAT
GGGGAATGGTTCCTTTATACTGGCAGTGGTGGAAGAGATCTGAGTGGAAACAAGCGCACG
AATAAATTTCAATCTTTTGACCAGAAGTTTGAAAACATGAATGAAGCTTTGAGACAAAGC
TGCCGAATGGGTTATCCTGTTCGTGTTGTAAGGTCGCATAAGGAAAAACGTTCTGCTTAT
GCACCCGAAGCTGGAGTGCGTTATGATGGGGTCTATAGAATAGAAAAATGCTGGCGCAAG
GATGGAATTCAAGGCTGTAAGGTTTGTAGGTATTTGTTTGTGAGGTGTGACAATGAGCCA

GCGCCGTGGACAAGTGATGAATCTGGAGACCGTCCACGACCACTGCCTGAGATTGTGGAG
TTAATTGGTGCAATTGATATAACAGAAAGGAAGGACGATCCATCGTGGGATTATGATGAA
GAAAAGGAGTGCTGGTTGTGGAAGAAGCCTCCTCCAGAAAGCAGGAAATCAGTGGACTCT
GGAGATGGTTCAACAGTCAGAGTTAAACGGAAGTCGAACATCGCGTCTGAGAGAGAAAAG
CTGCTGAAAGGGTTTCGTTGCGGAATATGCCGCAAGGTGATGCATTTACCCATTACTACA
CCTTGTGGTCACAACTTCTGCAAAGCCTGTTTGGAGAGTGCCTTTGCCGGACAAAGTTTG
ATGAGGGAAAGGTCACGTGAAGGTGGACGCAGTTTGCGAGCACAAAAGAATGTTAAGAAA
TGCCCATCATGTTCTAATGACATTGCTGACTTTCTTGAGAATCCACAGGTTAATAGAGAA
ATGCTGACTTTGATAGAATCATGGGGAAAGAAGCCTGAAGAAGAAGAAAACCCTGAGGAG
TCAAATGAAAATGATGAAAATGATGATAATGAAGAGAGTCAGGATGATGCAGCTGAGGTT
TCAAAACCTTCTGATTCTGCTGATAAGGTCCTGGAGGAAATTAAGGACAATGATCTGAAT
CAGCAACGTAAGCGAAGGAAGGAGTGA

>TRINITY_DN100132_c26_g1 (UNG_11)

ATGATATCAAGAGCAAATATGGAAGTGGGAGTCTCAAGAGTTACCAATGCCCATCACAAT
GCTCGAGGAGATGCAACAAGACCCAATACCACAAGCCCTGCATGTTTTTCTGTCAGAAGT
GCTGCAGGAAGTGCCTGTGTGTTCCTCCGGGTTATTATGGTAATAAGGCTGTGTGCCCTT
GCTACAACAACTGGAAGACCAAGGAGGGAGGACCCAAGTGCCCTTGAGCTTCAACCTCAA
CATCATATGTTGCTTTCTTATATATTTATCACTTCCTTCATTGTTCCATTTAAGGCACTA
GCTTACTTCCCTACTATTCACATGTCCTAA

>TRINITY_DN100144_c17_g1 (UNG_12)

ATGTCTCCTTCTCTCTCGCAAGCCAAATCTCTTCGTCTCCTTCTTCCTTTTCATCGGCGA
GGTTATGCAGTTGCGTCTGATGTTTCGGCAAGAGTTGGATTGGGTAATAATGTTGGGCGT
AGGAGTGGAATCGTGGGAGGTGCAGAAGAAAAGCCCGTGACAAGAGATGGTGCAAAAGCG
TATTCCGATTGGGCCCCAGACTCAGTAACCGGTGACTACAGGCCCATCAACCACACCCCT
GAAATTGACCCGGTGGAGCTCCGACGGATGTTGCTTAAGCACAAGTTCAAATCAGCACAG
TAG

>TRINITY_DN107844_c44_g1 (UNG_13)

ATGGAGGGGAAGGAGCAGGATGTGTCGTTGGGAGCGAACAAGTTCCCAGAGAGACAACCG
ATTGGTACGGCGGCGCAGAGCCAAGACGACGGCAAGGACTACCAGGAGCCACCGCCAGCG
CCGCTTTTTGAGCCCTCTGAGCTTACGTCGTGGTCGTTCTACAGAGCAGGGATAGCAGAG
TTTGTAGCAACTTTTCTGTTTCTCTACATCACCATCTTAACAGTCTTCGGTGTCCTTAGG
TCTCCCACCAAGTGCCAATCCGTTGGTATTCAAGGAATCGCTTGGGCCTTCGGTGGCATG
ATCTTCGCCCTCGTTTACTGCACCGCCGGAATCTCAGGGGGTCACATAAACCCTGCGGTT
ACCTTTGGGCTGTTCTTGGCAAGGAAATTGTCGTTGACCAGAGCGTTGTTCTACATCGTG

ATGCAAGTGTTGGGTGCTATCGCTGGCGCTGGAGTGGTGAAGGGTTTCGAGGGAAAAACC
AGATACGGTGCATTGAAAGGTGGTGCCAACTTTGTTAACCCAGGTTACACAAAAGGTGAC
GGACTCGGTGCTGAAATCGTTGGCACCTTTGTCCTTGTTTACACCGTTTTCTCAGCCACC
GATGCCAAACGTAGCGCCAGAGACTCTCACGTCCCCATTCTGGCACCTTTACCAATTGGG
TTCGCCGTGTTCTTGGTTCACTTGGCCACCATCCCCATCACCGGAACCGGTATCAACCCT
GCTCGTAGTCTCGGTGCTGCCATCATCTTCAACAAAGACCTTGGCTGGGACGATCACTGG
ATCTTCTGGGTGGGACCATTCATCGGTGCGGCTCTCGCTGCCCTCTACCACCAGGTCGTA
ATCCGCGCCATTCCCTTCAAGTCGAAGTGA
>TRINITY_DN127206_c45_g1 (UNG_14)
ATGGCAATGAAGAAGCTCTTGTGGGTTTTTCTGTCTCTTTCTTTGGTTCTTGGAGTGGCC
AATAGCTTAGATTTTCATGAGGAGGATTTGGCGTCCGAGGAAAGGTTGTGGGACTTGTAC
GAGAGATGGAGGAGTCGCCACACGGTTTCGCGAAGCCTCAGTGAGAAGCACAAACGGTTT
AACGTGTTCAAAGCAAATGTTATGCATGTGCATAACACTAACAAGATGGATAAGCCTTAC
AAGCTGAAACTGAACAAGTTCGCTGACATGACCAACCATGAATTTATGAGTACCTATGGA
GGCTCAAAGATTGATCACCATAAAATGTTCCGAGGCACGCAACATGGGAGCGGGACTTTC
ATGTATGAGAAGGTTGGGAGTGTTCCTGCTTCAGTGGATTGGAGAGAGAAAGGTGCTGTC
ACTAATGTGAAAAATCAGGGCCAATGTGGTAGCTGCTGGGCGTTTTCAGCTGTTGCAGCA
GTTGAAGGCATTAACCAAATAAAGACAAATAATTTGGTGTCATTGTCTGAACAAGAGTTG
ATCGATTGTGACAAAAAAGACAGCGGTTGTGAAGGTGGGTTGATGGAATCTGCTTTTGAG
TTCATCAAACAAAGAGGCATAACAACAGAAAGCAAATATCCTTACACAGCACAAGATGGA
CAGTGTGATGAATCAAAGGCGAACGACATATCTGTGTCAATTCAAGGTCATGAAAATGTC
CCTGCAAACGATGAAAATGCACTGCTCAAAGCTGTCGCCAACCAACCTGTTTCTGTAGCC
ATTGATGCTGGGGGATCTGATTTCCAGTTTTACTCCGAGGGAGTGTTTACTGGTCACTGT
GGCACGCTTCTAAATCATGGTGTAACGATTGTGGGATATGGAACAACGGTTGATGGGACT
AATTATTGGATAGTGAAAAACTCTTGGGGATCAGAATGGGGAGAACTTGGTTACATCAGA
ATGGAAAGGAACATATCTAACAAAGAGGGACTTTGCGGCATAGCCAAGGAGCCTTCCTAC
CCAATAGGATCTTTCTCATCTCCCAGAGATGAACTATGA
>TRINITY_DN59_c13_g1 (UNG_15)
GGCGGTGCAGTAAACGAGGATGAAGATCATGCCACCGAAGGCCCAAGCGATGCCCAAAAT
GCCTACCCCATCGCACTCGGAGGTACCGGCAACATTGGGGTCGGTCTGTCTCTTGTAGCC
GATAATGGTCAAGACGGTGATGTAAAGGAAGAGGAGGGTGGCTATGAATTCGGCTATGGC
GGCTCTGTACAAGGACCACTTGGTGAGCTCATCGGGGTCGAACAACGGCGCCGGAGGCGG
GTCGTGGTAGTCCTTGGCGGAGTATTCGGGTTGCTCCGTGACCTGCTCAACGTCTTTGGC
CATTGGAAGAAGGAATGCAGGAAAAGAGTTGCAGAGGAGAAGAGAGTAGTGTAG

>TRINITY_DN56042_c11_g1_i1.p1 HSP70

ATGGCAGCAAAGGAAGGCAAAGCAATAGGCATAGACCTCGGCACGACCTACAGCTGCGTA
GGCGTCTGGCAAAACGACCGCGTTGAGATCATCCCCAACGACCAAGGCAACCGAACCACA
CCCTCTTATGTGGCTTTCACCGACACCGAGAGGCTCATCGGAGACGCTGCCAAGAACCAA
GTTGCTATGAATCCGCAGAACACCGTCTTCGATGCTAAGCGTCTAATCGGGCGCAGATTC
TCCGACTCGTCGGTTCAAAACGACATGAAGCTATGGCCGTTTAAGGTGGTGGCGGGCCCG
GGTGACAAGCCCATGATCGTGGTCACGTACAAGGGTGAGGAGAAGAAGTTTTCTGCTGAA
GAGATATCTTCCATGGTGTTGATCAAGATGAGGGAAGTGGCAGAGGCGTTTCTTGGTCAT
TCCGTGAAGAACGCTGTTATTACTGTCCCTGCTTATTTCAACGACTCGCAAAGGCAAGCC
ACGAAAGACGCAGGGGCCATTTCAGGGTTGAATGTGTTGAGAATCATCAACGAACCCACT
GCCGCTGCTATTGCCTACGGCTTGGACAAGAAGGCTTCAAGAAAAGGTGAACAGAACGTG
CTTATTTTCGACTTGGGTGGTGGAACCTTTGATGTTTCCATATTGACCATCGAGGAGGGG
ATTTTCGAAGTGAAGGCCACTGCTGGTGATACCCATCTCGGAGGTGAAGATTTTGATAAC
AGGTTGGTGAATCACTTTGTTACAGAGTTCAAAAGGAAGCACAAAAAGGATATTAGCGGG
AATGCTAGAGCGTTGAGGAGGTTGAGGACAGCCTGTGAGAGAGCGAAGAGGACGTTGTCT
TCCACCGCGCAGACAACCATTGAAATCGATTCTTTATACGAAGGGATTGATTTCTATGCA
ACGATTACCAGAGCCAGGTTTGAGGAGATGAACATGGACTTGTTCAGGAAGTGCATGGAG
CCGGTGGAGAAGTGTCTGCGTGATGCCAAGATTGACAAGAGTCACGTTCATGAAGTTGTT
CTTGTTGGAGGGTCCACCAGGATCCCGAAGGTTCAGCAACTGTTGCAGGATTTCTTCAAC
GGGAAAGAGCTTTGCAAGAGCATTAACCCCGACGAAGCTGTTGCTTATGGTGCAGCTGTT
CAGGCTGCGATTTTGAGTGGTGAAGGGGACGAGAAGGTTCAGGATTTGTTGCTGTTGGAT
GTGACTCCACTCAGTCTTGGGCTTGAAACTGCTGGTGGTGTTATGACAGTGTTGATTCCG
AGGAACACAACGATTCCGACCAAGAAGGAGCAGATTTTCTCGACTTATTCCGATAACCAA
CCCGGGGTTTTGATCCAAGTGTTTGAAGGAGAGCGTGCTCGAACAAAGGACAACAATCTT
CTCGGGAAGTTTGAGCTCACTGGGATCCCTCCTGCACCAAGAGGCGTTCCTCAGATTAAT
GTCTGCTTCGACATTGATGCTAATGGGATTTTGAATGTCTCTGCTGAGGATAAAACTGCG
GGTGTGAAGAACAAGATTACTATAACAAACGACAAGGGTAGGTTGAGCAAGGAGGAGATA
GAGAAGATGGTGAAGGATGCAGAGAGGTACAAGGCAGAGGATGAAGAGGTGAAGAAGAAG
GTGGACGCGAAAAACTCGCTTGAGAATTATGCTTACAACATGAGGAACACGATAAAGGAT
GAGAAGATTGGGGGGAAGTTGGGGGCGGATGATAAGCAGAAGATTGAGAAGGCTGTGGAG
GATGCGATTCAGTGGCTGGAGGCGAACCAGTTGGCGGAAGTGGATGAGCTTGAAGACAAG
CAGAAGGAGTTGGAAGGAATCTGCAACCCCATCATTGCCAAGATGTATCAGGGTGGTGCC
GGTGGAGATGTTCCTATGGCTGATGACATGCCTGGTGGTGGGTCTGGTTCTGGTGCTGGA
CCTAAGATTGAAGAAGTTGACTGA

>TRINITY_DN100383_c0_g1_i1.p1 Desiccation-related_protein_PCC13-62

ATGCATCATCATTTTGAAAAAAAAAAAGAGCCTGAAATGGCACCTCGCAATTCCAGAGTC
AGAGTCTTTTTTGCTGTTTTGGTTGCTTCCCTAGTCCTTCCCTTGTTTTTCCCAGAATGT
TCTTCTTCTTCTGTGCTCATTGCAAGAGCATCAGCCCCAAAATCAGATATTGATCTTTTA
GAATTCCCTCTAAACTTAGAATACTTGGAGGCTGAATTCTTCTTGTTTGGAGCTTTGGGT
CATGGATTGGATGTGTTTGCTCCAAAACTGGCCGGGGGAGGACCTCCTCCCATTGGTGCC
AAAGCTGCCAACCTCGACAACTTTTTTAAGGATGTCATATTGCAGTTTGGTTTGCAAGAA
GTCGGACACTTGAGGGCTATAAAGAGCACAGTGAAAGGATTCCCTAGGCCTCTGCTGGAT
CTAAGCCCTTCATCTTTTGCCAAAGTAATGGATAATGCGGTTGGGAGAACTCTGAGTCCA
CCCTTTGACCCCTATGCTAATTCAATCAACTTTCTTCTTGCTTCTTACGTTATTCCCTAT
GTTGGCCTCACTGGCTATGTTGGTGCCAATCCAAAGCTGCAAAATGCTACTTCCAGGGAG
CTTGTGGCAGGGCTGCTAGGAGTAGAATCAGGACAAGATGCAGTTATAAGGGCATTTTTG
TATGAACGGAAAAGGCAATTGGTGCATCCGTATGGATTGAGCGTGGGAGAGTTCGCAGAT
CGTATTTCAAATCTGAGGAACAAGCTAGGAAAAGAAGGTTTGAAAGATGAGGGTCTTGTG
GTTGCTAAGGAGTATGGTGCTGAGAAGGAAGTGAATGGGAACATTCTGGCTGGTGACAGA
GATTCACTGGCATATTCAAGAACCCCAGAGGAAATATTGAGGATAATATATGGAACAGGT
GATGAACATGTTCCTGGTGGCTTCTACCCTAAAGGAGCAAGTGGTCGCATAGCAAGACTT
TACTTGAAGCATTCCGTCTAA

>TRINITY_DN100101_c9_g1_i1.p1 flower_spec_ACCoxidase (ACC)

ATGGCAAACTTTCCAGTTGTTGACATGGGAAAGCTTAACACTGAAGAGAGAGGAGCTGCC
ATGGAAAGGATAAAAGATGCTTGTGAGAACTGGGGTTTCTTTGAGTTGGTGAACCATGGT
ATATCCATTGAGTTGATGGACACCGTGGAGAAGTTAACAAAAGAGCACTACAAGAAGACT
ATGGAGCAAAGGTTCAAAGAAATGGTGGCCAACAAAGGTCTTGAGTCAGTTCAGTCAGAA
ATCAATGACTTGGACTGGGAAAGTACCTTTTTTCTGCGCCATCTTCCAGTCTCCAATGTT
TCAGAGAACACAGATCTTGATGAAGACTACAGGAAGATAATGAAGCAGTTTGCAGAAGAA
CTGGAGAAACTTGCAGAGCATCTTCTTGACTTGCTGTGTGAGAATCTTGGACTGGAGAAA
GGGTACCTGAAGAAGGTGTTCTATGGATCGAAGGGCCCAAATTTTGGCACGAAAGTTAGC
AACTACCCTCCTTGTCCGACCCCTGATCTGATAAAGGGCCTAAGAGCTCACACTGATGCC
GGTGGCATTATCCTACTGTTCCAAGATGACAAGGTCAGTGGACTGCAGCTCCTCAAGGAT
GACCAGTGGATCGATGTCCCACCAATGCGTCACTCCATTGTCATCAACCTTGGTGACCAA
CTTGAGGTCATAACCAATGGCAAGTACAAGAGTGTCATGCACCGAGTCATTGCTCAGACC
GATGGCACCAGAATGTCCCTGGCTTCCTTCTATAATCCCGGTGATGATGCTGTGATTTCT
CCAGCACCAGCCTTGGTGAAGGAATCGGATGAAACAAGCCAAGTATACCCGAAATTTGTG
TTTAATGATTACATGAAGCTCTATGCTGGTCTCAAGTTTCAGGCTAAAGAACCAAGGTTT

GAAGCTATGAAGGCCGTGTCAAGCGTTGATGTGGGGGCCATAGCCACAGTTTGA

>TRINITY_DN1811_c6_g1_i1.p1flower_spec_gibberellin_regulated_protein (F-GP)

ATGGCTCTTTCCAAGCTCATACTCGCTTCCCTTCTCGCCTCGCTTCTCCTGCTTCATCTC

GTTGATGCTAATCAATCGGTGCAAGCACAGACGCAGGGTTCTCTTCTTCAGAAGATAGAC

TGTAACGGAGCATGTGCTGCGAGATGCCGTTTATCATCTCGTCCTCGTCTCTGCAAAAGA

GCTTGTGGAACTTGTTGCAGTCGCTGCAACTGCGTCCCACCTGGTACTTCTGGAAACCAA

GAAACGTGTCCCTGCTATGCTAGCTTAACTACTCATGGTGGCAAACGCAAGTGCCCTTAA
