# Supplementary figures and images for: De novo transcriptome assembly and analysis of gene expression in different tissues of moth bean (Vigna aconitifolia) (Jacq.) Marechal
Source: BMC Plant Biol. 2022 Apr 15;22:198. doi: 10.1186/s12870-022-03583-z (PMC9013028; doi:10.1186/s12870-022-03583-z)

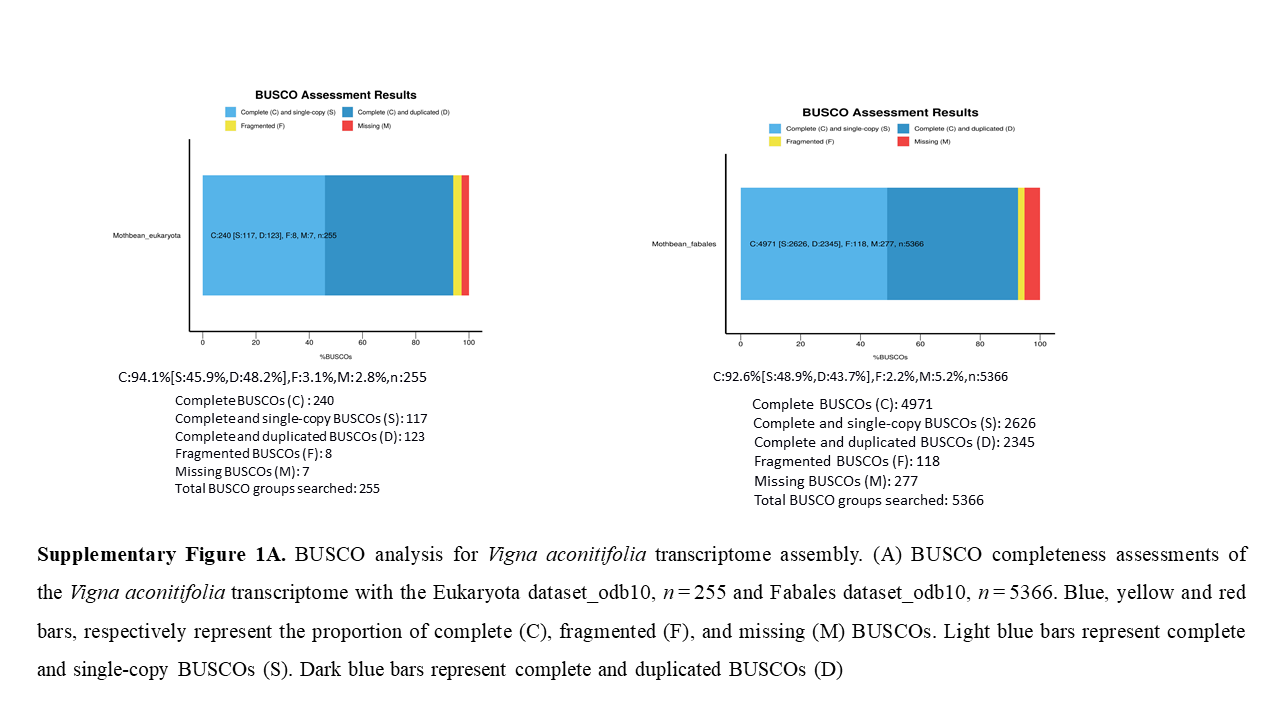

Supplement: Supplementary file 2 — Additional file 2: Supplementary Fig 1a. [file 12870_2022_3583_MOESM2_ESM.tif]

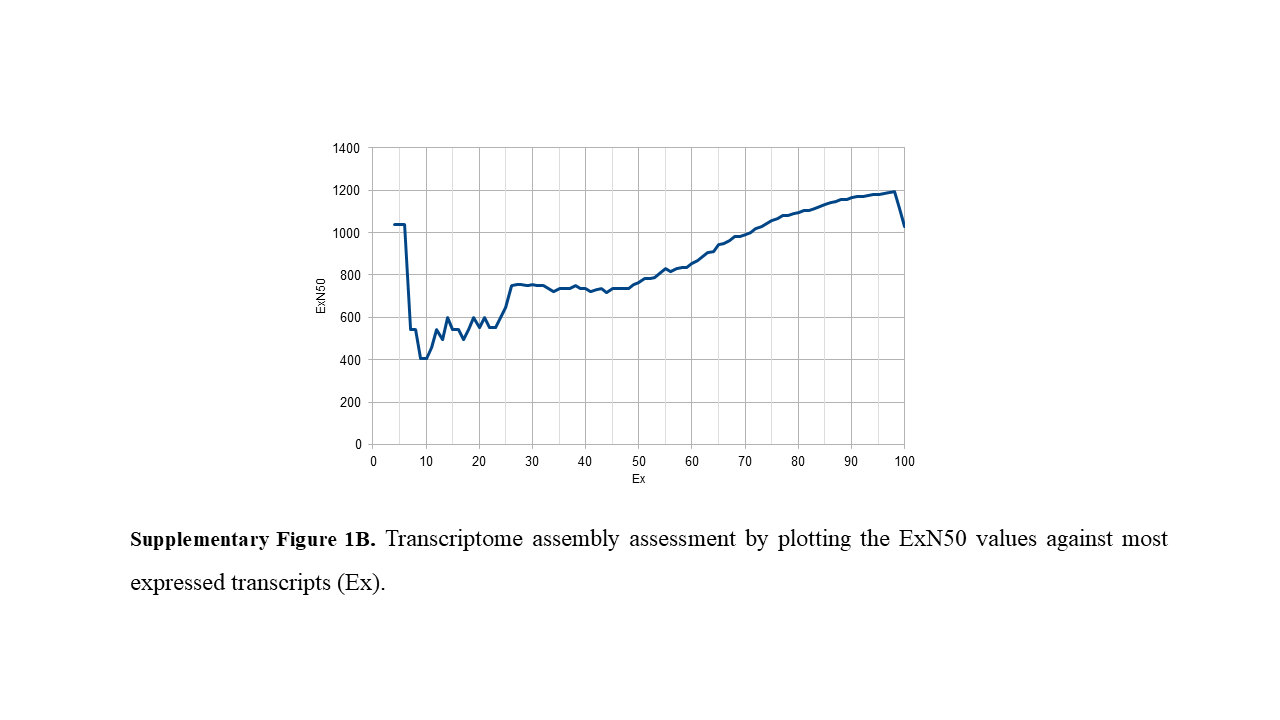

Supplement: Supplementary file 3 — Additional file 3: Supplementary Fig 1b. [file 12870_2022_3583_MOESM3_ESM.tif]

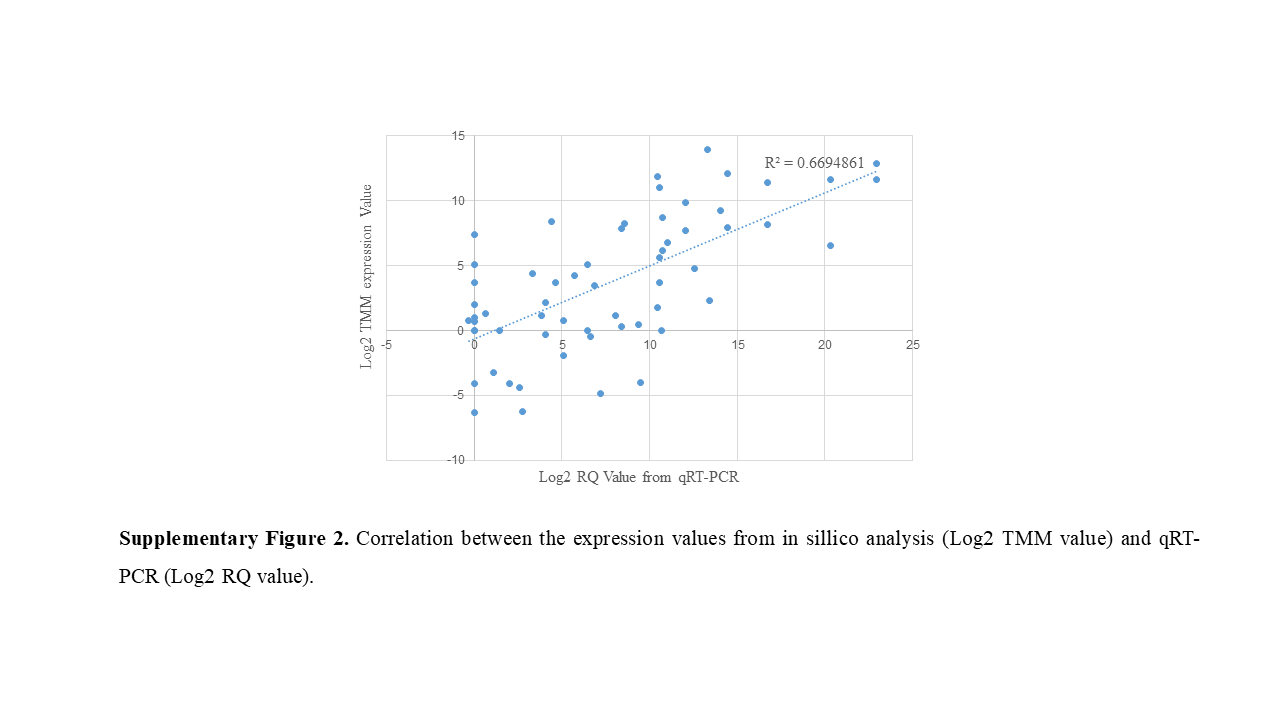

Supplement: Supplementary file 4 — Additional file 4: Supplementary Fig 2. [file 12870_2022_3583_MOESM4_ESM.tif]
